# Uncommon Manifestations of Lung Cancer: Diagnostic Challenges and Valuable Insights From a Rare Clinical Case

**DOI:** 10.7759/cureus.71249

**Published:** 2024-10-11

**Authors:** Kristina Petrova, Lyubomir Gaydarski, Radoil Simeonov, Lubomir Tsvetanov, Boycho Landzhov, Georgi P Georgiev

**Affiliations:** 1 Department of Clinical Laboratory, Medical University of Sofia, Sofia, BGR; 2 Department of Anatomy, Histology and Embryology, Medical University of Sofia, Sofia, BGR; 3 Department of Orthopaedics and Traumatology, University Hospital Queen Giovanna - ISUL, Sofia, BGR

**Keywords:** acrometastasis, bone metastasis, diagnostics, lung cancer, untypical manifestation

## Abstract

Lung cancer is commonly diagnosed at advanced stages, often presenting with metastases. Although bone metastases are common in lung cancer patients, acrometastases - metastatic lesions in the bones of the hand - are exceedingly rare. Herein, we report the case of a 71-year-old male with previously undiagnosed lung adenocarcinoma, which first manifested as a painful swelling in the right hand. Radiographic imaging and biopsy revealed a bone metastasis involving the third metacarpal and phalanges, secondary to lung adenocarcinoma. Three weeks after the biopsy, the hand tumor became severely ulcerated, leading to a significant drop in hemoglobin levels, necessitating an urgent amputation as a life-saving measure. This case highlights the diagnostic challenges of rare metastatic patterns and emphasizes the need for timely and accurate diagnosis to improve outcomes. Clinicians should consider metastases in the differential diagnosis of unexplained hand swelling, and early intervention is critical in managing such aggressive cases.

## Introduction

Lung cancer is one of the most common and aggressive forms of cancer, with a high mortality rate [1]. It is classified into several subtypes, with non-small cell lung cancer (NSCLC) being the most prevalent. This category includes adenocarcinoma, squamous cell carcinoma, and large cell carcinoma, which together account for 80% of all lung cancer cases [2]. Typical clinical symptoms include cough, hemorrhagic sputum, chest pain, shortness of breath, dyspnea, fatigue, weight loss, etc. [3]. Lung cancer often causes metastasis with typical sites, including the lungs, liver, and bones, with the axial skeleton - vertebrae, ribs, sternum, and pelvis - being particularly affected due to their higher bone marrow content [1,4]. A significant number of cases are diagnosed in the later stages when metastases are already present [4]. Acrometastases, which are metastatic lesions in the hands and feet, are extremely rare, with lung cancer being the most common origin, followed by prostate and breast cancer [5]. In sporadic cases, acrometastases can be the initial presentation of stage IV lung cancer, with a survival rate of approximately six to seven months [4,6]. Over 30% of lung cancer patients develop bone metastases, with adenocarcinoma being the subtype most likely to spread to the bones [7]. The incidence of bone lesions in the upper extremities ranges from 10% to 15%, with only 0.1% occurring in the hand [8-10]. The distal phalanges are the most frequent site of hand metastases [11]. Specifically, 66% of hand metastases occur in the phalanges, 17% in the metacarpal bones, and 17% in the carpal bones [11]. Bone metastases are a sign of a more aggressive cancer and are associated with a worse prognosis [6]. Common symptoms include pain, local swelling, increased circumference of the affected finger, and limited movement [8]. The differential diagnosis includes rheumatoid arthritis, ingrown toenails, osteomyelitis, osteoarthropathy, gout, reflex sympathetic dystrophy, and fractures [12]. Diagnosis is often delayed, negatively affecting the patient's quality of life [8]. X-rays are crucial in diagnosis, frequently revealing osteolytic lesions [8]. Other diagnostic tools include computed tomography (CT), magnetic resonance imaging (MRI), positron emission tomography (PET), and thin needle biopsy [8]. Amputation is the most common surgical approach due to the short life expectancy [8]. In this report, we present the case of a 71-year-old male with previously undiagnosed lung adenocarcinoma, which first manifested as bone metastases in the third metacarpal joint and the proximal and middle phalanges of the third finger. We also provide a brief literature review and discuss diagnostic and treatment approaches.

## Case presentation

A 71-year-old male presented with swelling of the right hand, specifically localized to the palmar region. The patient reported that the swelling had developed gradually over three months, with no history of trauma. Initially asymptomatic, the patient later experienced a constant dull pain in the wrist and hand. The swelling progressively increased in size, ultimately impairing the function of his dominant right hand and interfering with daily activities. The patient reported no relevant medical history or known allergies and only mentioned that he has lost a significant amount of weight in the past three to four months, around 25 kg. The patient reported no history of smoking or alcohol use. On physical examination, swelling was observed on the palmar surface, primarily in the region overlying the head of the third metacarpal bone, extending to the third finger up to the distal phalanx. Palpation revealed a tense mass, soft on the exterior but firm internally. Flexion of the third finger was entirely lost, and flexion of both the index and ring fingers was also impaired. Blood tests showed normal values, which are summarized in Table 1.

**Table 1:** Laboratory results before the biopsy. HGB: hemoglobin; HCT: hematocrit; RBC: red blood cells; WBC: white blood cells; GRAN: granulocytes; EO: eosinophils; BAS: basophils; LYM: lymphocytes; MON: monocytes; PLT: platelets; ALT: alanine aminotransferase; AST: aspartate aminotransferase; ALP: alkaline phosphatase; CRP: C-reactive protein

Parameters and units	Results	Reference ranges (male)
HGB (g/L)	137	140-180
HCT (L/L)	0.42	0.37-0.55
RBC (10*12/L)	4.34	4.2-6.2
WBC (10*9/L)	6.7	3.5-10.5
GRAN%	72	44-76
EO%	1.15	0-6
BAS%	0.4	0-2
LYM%	18.2	20-40
MON%	5.1	3-13
PLT (G/L)	280	130-440
ALT (U/I)	19	0-42
AST (U/I)	14	0-41
ALP (U/I)	39	20-140
CRP (mg/dL)	0.67	0-0.6
Albumin (g/L)	39	35-50
Total protein (g/L)	68	63-84
Glucose (mmol/L)	6.48	3.3-6
Potassium (mmol/L)	3.6	3.5-5.1
Sodium (mmol/l)	138	135-145
Chloride (mmol/L)	105	98-107
Creatinine (µmol/L)	67	50-133
Urea (mmol/L)	4.5	1.7-8.30

**Figure 1:**
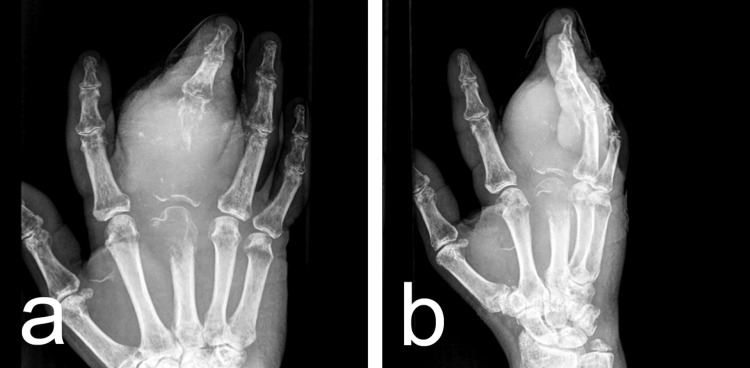
An initial X-ray of the swollen hand revealed a large soft tissue mass surrounding the head of the third metacarpal bone, as well as the proximal and middle phalanges of the third finger, accompanied by bone lysis.

Radiographic imaging of the hand demonstrated a significant soft tissue mass surrounding the head of the third metacarpal bone and the proximal and middle phalanges of the third finger, accompanied by bone lysis (Figure 1).

**Figure 2:**
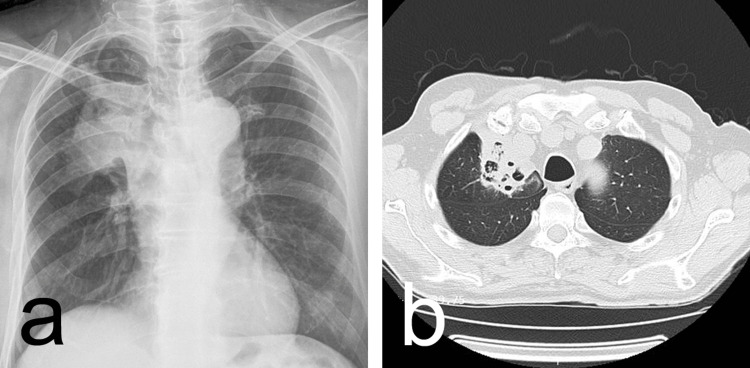
(a) An X-ray revealing diffuse opacity indicative of an infiltrative process in the medial aspect of the right lung's upper lobe, just above the hilum; (b) Computed tomography revealing a diffuse infiltrative process in the medial portion of the right upper lobe, with pleural involvement.

A chest X-ray revealed diffuse opacity indicative of an infiltrative process in the medial aspect of the right lung's upper lobe, just above the hilum (Figure 2a). Subsequent full-body imaging confirmed a diffuse infiltrative process in the medial portion of the right upper lobe, with pleural involvement (Figure 2b).

**Figure 3:**
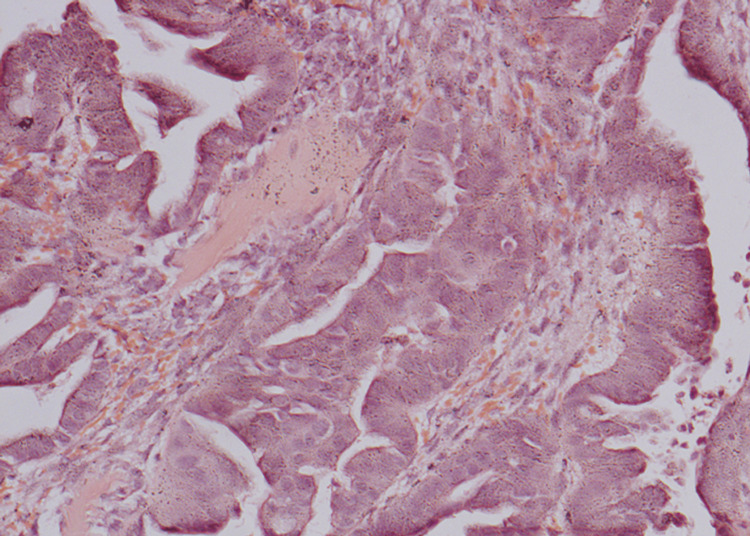
Histopathological image showing metastatic bone infiltration, highly suggestive of adenocarcinoma of pulmonary origin (hematoxylin and eosin staining at 20X magnification).

An open biopsy of the hand mass was performed using a radial approach. Histological examination showed metastatic bone infiltration, highly suggestive of adenocarcinoma of pulmonary origin (Figure 3).

Three weeks after the biopsy, the patient's condition deteriorated. The hand tumor exhibited rapid growth, accompanied by a significant decrease in hemoglobin levels and systemic signs of toxic syndrome (Table 2).

**Table 2:** Laboratory results before the surgery. HGB: hemoglobin; HCT: hematocrit; RBC: red blood cells; WBC: white blood cells; GRAN: granulocytes; EO: eosinophils; BAS: basophils; LYM: lymphocytes; MON: monocytes; PLT: platelets; ALT: alanine aminotransferase; AST: aspartate aminotransferase; ALP: alkaline phosphatase; CRP: C-reactive protein; TIBC: total iron-binding capacity; CK: creatine kinase; aPTT: activated partial thromboplastin time; INR: international normalized ratio

Parameters and units	Results	Reference ranges (male)
HGB (g/L)	93	140-180
HCT (L/L)	0.25	0.37-0.55
RBC (10*12/L)	2.77	4.2-6.2
WBC (10*9/L)	8.79	3.5-10.5
GRAN%	66.41	44-76
EO%	0.27	0-6
BAS%	0.6	0-2
LYM%	24.63	20-40
MON%	8.09	3-13
PLT (G/L)	227	130-440
ALT (U/I)	34	0-42
AST (U/I)	20	0-41
ALP (U/I)	115	20-140
CRP (mg/dL)	6.83	0-0.6
Albumin (g/L)	40	35-50
Total protein (g/L)	67	63-84
Glucose (mmol/L)	8.29	3.3-6
Potassium (mmol/L)	4.4	3.5-5.1
Sodium (mmol/l)	144	135-145
Chloride (mmol/L)	104	98-107
Serum Calcium (mmol/L)	2.17	2,15 – 2,5
Ionized Calcium (mmol/L)	1.16	1.20- 1.40
Magnesium (mmol/L)	0.9	0,66-1,07
Iron (µmol/L)	5.1	5.83-34.5
TIBC (µmol/L)	30.4	45-75
CK (U/L)	120	39-308
Creatinine (µmol/L)	88	50-133
Urea (mmol/L)	5	1.7-8.30
aPTT (sec.)	28.1	23-31.9
INR (INR)	1.23	0.85-1.25
Fibrinogen (mmol/L)	20	<7,1

**Figure 4:**
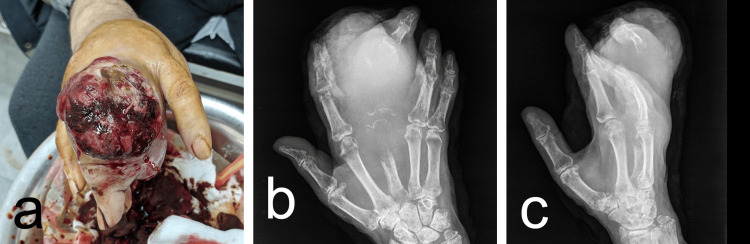
(a) Preoperative image showing the necrotic surface of the tumor with hemorrhagic exudate; (b, c) Preoperative X-rays demonstrating enlargement of the soft tissue mass surrounding the head of the third metacarpal bone and the proximal and middle phalanges of the third finger, extending to involve the adjacent second and fourth fingers, and accompanied by bone lysis.

The anatomy of the hand became grossly distorted, and the surface of the tumor showed necrosis, with hemorrhagic exudate leaking from the biopsy site (Figure 4).

**Figure 5:**
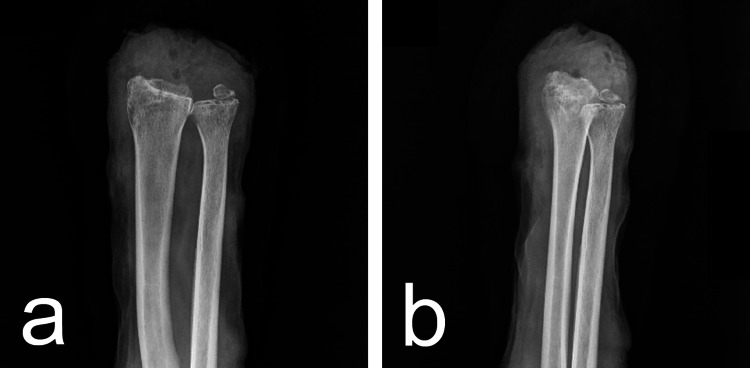
(a, b) Postoperative X-ray of the forearm.

An urgent life-saving surgical intervention was undertaken. Under general anesthesia and with a tourniquet applied, the hand amputation was performed. The patient was positioned supine, with the right hand in supination. Skin incisions created volar and dorsal flaps in a 2:1 ratio, extending 1.3 cm from the ulnar and radial styloid processes. Dissection of the underlying soft tissues was carried out, with ligation of subcutaneous veins. The radial and ulnar arteries were identified and ligated, along with the flexor and extensor tendons, which were dissected and severed. The ulnar, median, and radial nerves were dissected, infiltrated with 2% lidocaine, and transected. The radiocarpal joint capsule and ligaments were incised, facilitating joint disarticulation. The remaining tendons of the flexor and extensor muscles were anchored to bone tunnels in the radius. Hemostasis was achieved, the wound was irrigated, and a subcutaneous suction drain was placed before closing the skin flaps (Figure 5). The surgical procedure was performed in accordance with the techniques described in *Campbell's Operative Orthopaedics, 14th Edition *[13].

The patient's condition improved postoperatively, and he was discharged from the department several days later with a referral to pulmonology and oncology for further diagnostics and management of the primary tumor.

## Discussion

Lung cancer is frequently diagnosed at an advanced stage, often presenting with metastatic lesions. Bone metastases occur in over 30% of patients with lung cancer, yet it is rare for asymptomatic lung cancer to manifest first through complaints related to bone metastasis [4]. Among these, metastases to the bones of the hand, or acrometastases, are particularly rare, accounting for only 0.1% of cases [8-10]. The hand bones most commonly affect the phalanges in approximately 66% of cases [11]. The rarity and nonspecific presentation of acrometastases often lead to diagnostic delays, significantly worsening patient prognosis [8]. According to Afrăsânie et al., the low occurrence of acrometastases could be due to the reduced amount of red bone marrow in distal bones [6]. When acrometastases typically affect the dominant hand, possibly due to increased trauma exposure and greater blood flow [6]. These metastases are more frequently diagnosed in men and usually occur between 40 and 80 years of age [6].

In the current case, we report a patient who presented with a three-month history of painful swelling in the right palmar region. A biopsy revealed that the swelling was caused by a metastatic lesion from a previously undiagnosed lung adenocarcinoma. This case underscores the diagnostic challenges of hand acrometastases and emphasizes the importance of considering metastasis in the differential diagnosis of unexplained painful hand swelling. Intriguingly, the patient reported no history of smoking or alcohol use, which did not assist in diagnosing potential cancer, as the typical risk factors were absent. Several other case reports reinforce this observation. For instance, Afrăsânie et al. described a case where the first sign of lung adenocarcinoma was painful swelling in the distal phalanx of the right index finger [6]. Rinonapoli et al. reported a case in which large-cell lung cancer metastasized to the carpal region of the left hand, initially misdiagnosed as tendonitis and osteoarthritis, emphasizing the importance of a thorough diagnostic evaluation and follow-up [14]. Houimli and Selmene also described a case where swelling in the first phalanx of the third finger was the initial manifestation of lung adenocarcinoma [15]. Similarly, Keramidas and Brotherston reported a rare case of extensive swelling in the right hand, involving the carpal, metacarpal, and phalangeal regions, as the first presentation of lung adenocarcinoma [16]. These cases highlight the unusual nature of lung cancer presenting with hand metastases and the frequent diagnostic delays that accompany it.

Clinically, hand metastases typically present as painful swelling with erythema. Due to their rarity, these lesions are often initially misdiagnosed as infections, further delaying the correct diagnosis [16]. Diagnostic evaluation usually begins with X-rays, which can reveal bone lesions or pathological fractures. Still, they have limited sensitivity, detecting bone destruction only after more than 50% of the bone has been compromised [17]. Radiographically, bone metastases may appear as lytic lesions, which are more common in lung cancer, or as sclerotic or mixed lesions, which are typically associated with breast cancer but can also occur in lung cancer. Osteoblastic lesions are most often seen in prostate cancer [8,17]. More advanced imaging techniques are usually necessary for an accurate diagnosis. CT is commonly used for whole-body imaging and offers higher sensitivity and resolution than X-rays in detecting bone structures [18]. MRI is valuable for evaluating soft tissue components and spinal cord involvement in metastatic lesions [19]. Although more sensitive than X-rays, bone scintigraphy has low specificity and a high rate of false positives [18]. PET-CT is the most sensitive modality, capable of detecting metastatic lesions earlier and distinguishing between benign and malignant findings [8]. PET-CT has a sensitivity exceeding 90% and a specificity of 78% [8]. Diagnosis is confirmed through histological analysis via a thin needle or open biopsy [9]. In our case, the biopsy led to severe deterioration of the patient's condition, highlighting the risk of rare but severe complications associated with this procedure.

The treatment of lung cancer with bone metastases can be systemic or local. First-line systemic treatment typically involves platinum-based chemotherapy, such as cisplatin, carboplatin, and oxaliplatin, while targeted therapies are preferred when specific molecular aberrations are present [20]. In our case, we managed the patient orthopedically and referred him to an oncologist and pulmonologist for cancer treatment. Bisphosphonates or denosumab are commonly prescribed for managing bone metastases, both of which act as antiresorptive agents that prevent further bone damage and help alleviate pain [20]. Local treatment options include radiotherapy and surgery. In severe cases, such as those involving intractable pain or when the patient's life expectancy exceeds five months, amputation may be considered the most effective surgical intervention [12]. For patients with a poorer prognosis, more conservative approaches, including radiotherapy combined with pain management, can effectively alleviate symptoms and improve function [12].

In this case, the rapid progression of the tumor and the development of toxic syndrome following the biopsy necessitated the amputation of the right hand as a life-saving measure. This case highlights the importance of recognizing the possibility of metastasis in atypical presentations, such as painful hand swelling. It demonstrates the challenges in both diagnosing and managing such rare manifestations of lung cancer.

## Conclusions

The present case underscores the critical need for heightened clinical awareness regarding rare metastatic presentations of lung cancer such as acrometastases. While bone metastases are relatively common in advanced lung cancer, the manifestation of metastases in the hand, particularly as the first clinical sign, is extremely rare. This rarity often leads to diagnostic delays, as in this case, where hand swelling and pain were initially attributed to more common benign conditions. Early recognition of such atypical presentations is crucial to prevent diagnostic delays, which can significantly worsen the patient's prognosis. Essentially, this case illustrates the complexity and challenges associated with diagnosing and treating lung cancer with unusual metastatic patterns, reinforcing the need for comprehensive diagnostic evaluations and personalized treatment strategies.

## References

[1] Wong MC, Lao XQ, Ho KF, Goggins WB, Tse SL (2017). Incidence and mortality of lung cancer: global trends and association with socioeconomic status. Sci Rep.

[2] D'Addario G, Früh M, Reck M, Baumann P, Klepetko W, Felip E (2010). Metastatic non-small-cell lung cancer: ESMO Clinical Practice Guidelines for diagnosis, treatment and follow-up. Ann Oncol.

[3] Xing PY, Zhu YX, Wang L (2019). What are the clinical symptoms and physical signs for non-small cell lung cancer before diagnosis is made? A nation-wide multicenter 10-year retrospective study in China. Cancer Med.

[4] Morris G, Evans S, Stevenson J, Kotecha A, Parry M, Jeys L, Grimer R (2017). Bone metastases of the hand. Ann R Coll Surg Engl.

[5] Spiteri V, Bibra A, Ashwood N, Cobb J (2008). Managing acrometastases treatment strategy with a case illustration. Ann R Coll Surg Engl.

[6] Afrăsânie VA, Adavidoaiei AM, Zamisnicu IH (2019). A very rare presentation of lung cancer: metastases to the distal phalanx of index-case report. Medicine (Baltimore).

[7] Zhang L, Gong Z (2017). Clinical characteristics and prognostic factors in bone metastases from lung cancer. Med Sci Monit.

[8] Afshar A, Farhadnia P, Khalkhali H (2014). Metastases to the hand and wrist: an analysis of 221 cases. J Hand Surg Am.

[9] Amadio PC, Lombardi RM (1987). Metastatic tumors of the hand. J Hand Surg Am.

[10] Kerin R (1987). The hand in metastatic disease. J Hand Surg Am.

[11] Basora J, Fery A (1975). Metastatic malignancy of the hand. Clin Orthop Relat Res.

[12] Healey JH, Turnbull AD, Miedema B, Lane JM (1986). Acrometastases. A study of twenty-nine patients with osseous involvement of the hands and feet. J Bone Joint Surg Am.

[13] Campbell WC, Canale ST, Beaty JH (2020). Major amputations of the upper extremity. Campbell's Operative Orthopaedics, Fourteenth Edition.

[14] Rinonapoli G, Caraffa A, Antenucci R (2012). Lung cancer presenting as a metastasis to the carpal bones: a case report. J Med Case Rep.

[15] Houimli S, Selmene MA (2020). Uncommon bone metastases of the hand. Open J Orthop.

[16] Keramidas E, Brotherston M (2005). Extensive metastasis to the hand from undiagnosed adenocarcinoma of the lung. Scand J Plast Reconstr Surg Hand Surg.

[17] Choi J, Raghavan M (2012). Diagnostic imaging and image-guided therapy of skeletal metastases. Cancer Control.

[18] Rosenthal DI (1997). Radiologic diagnosis of bone metastases. Cancer.

[19] Evans AJ, Robertson JF (2000). Magnetic resonance imaging versus radionuclide scintigraphy for screening in bone metastases. Clin Radiol.

[20] Masters GA, Temin S, Azzoli CG (2015). Systemic therapy for stage IV non-small-cell lung cancer: American Society of Clinical Oncology clinical practice guideline update. J Clin Oncol.

